# Acceptance and Commitment Therapy for Obesity

**DOI:** 10.1007/s13679-025-00634-y

**Published:** 2025-05-10

**Authors:** Leah M. Schumacher, Nicole Miller, Emma L. Jennings, Reena Chabria, Meghan L. Butryn

**Affiliations:** 1 Department of Social and Behavioral Sciences, College of Public Health, Temple University, Philadelphia, PA USA; 2 Center for Obesity Research and Education, College of Public Health, Temple University, Philadelphia, PA USA; 3 Center for Weight, Eating, and Lifestyle Science, Drexel University, Philadelphia, PA USA; 4 Department of Psychological and Brain Sciences, Drexel University, Philadelphia, PA USA

**Keywords:** Obesity, Overweight, Acceptance and Commitment Therapy, Weight Stigma, Weight Loss, Adherence

## Abstract

**Purpose of Review:**

To describe the recent literature on acceptance and commitment therapy (ACT) interventions for individuals with obesity. The review begins with a brief overview of the ACT model, describes seminal work in this area, and examines more recent literature on the use of ACT to improve outcomes among individuals with obesity.

**Recent Findings:**

Early trials established ACT’s efficacy for weight loss among adults with obesity. More recent research has focused on testing efficacy among adolescents, measuring effects in “real world” settings, refining interventions to optimize outcomes and enhance scalability, and examining outcomes beyond weight (e.g., internalized weight stigma, eating regulation). Current data indicate that ACT-based interventions produce comparable, or, in some cases, superior weight loss compared to standard behavioral interventions. ACT has also shown promise for improving other outcomes of interest.

**Summary:**

ACT may improve a variety of obesity-related outcomes, although additional research is needed.

## Introduction

One in eight individuals worldwide live with obesity, as defined by a body mass index (BMI) of ≥ 30.0 kg/m^2^ [1]. Obesity is a multifactorial disease with diverse, multi-level contributors [1, 2]. As such, many efforts, ranging from national policies to individual health initiatives, have been directed toward preventing and treating obesity [2].

At the individual level, comprehensive lifestyle intervention, also called behavioral treatment, remains a recommended treatment option [3, 4]. This approach prescribes changes in diet and physical activity and teaches strategies that facilitate behavior change [5]. Modification of eating behaviors and physical activity is also important for patients pursuing pharmacotherapy or metabolic bariatric surgery [3, 6]. Regardless of treatment approach, many patients have difficulty making changes to weight-related behaviors and sustaining these changes over time [7, 8]. Some patients may also experience suboptimal weight loss or weight recurrence (i.e., regain) despite successfully making behavioral changes due to biological adaptation and other factors [9, 10]. Additionally, many individuals experience weight-related psychosocial difficulties, such as distress due to anticipated or experienced weight stigma (i.e., prejudice or discrimination based on body weight), internalized weight stigma (also referred to as weight self-stigma and internalized weight bias, among other terms; i.e., self-devaluation or the application of negative stereotypes about weight to oneself), and body image concerns [11–15]. Together, these factors can cause distress and make the long-term treatment of obesity challenging, including by contributing to weight gain and impeding health behavior engagement [13–15].

Over the past two decades, acceptance and commitment therapy (ACT) has emerged as a promising treatment approach for those with obesity. ACT has been used both to enhance behavior change and weight loss outcomes from individual-level, behavioral interventions, as well as to improve difficulties often associated with obesity.

In this review, we provide a high-level overview of ACT, highlight seminal studies investigating the utility of ACT for obesity, and review recent research on the potential benefits for individuals with obesity, with a focus on weight and selected non-weight outcomes. Due to the size of the literature, this review focuses primarily on studies that have used ACT as part of comprehensive obesity lifestyle modification interventions (i.e., targeting eating and physical activity) or those enrolling only individuals with overweight or obesity. Additionally, our discussion encompasses ACT’s impact on several non-weight outcomes including body-related concerns, internalized weight stigma, and facets of eating regulation. ACT’s impact on dietary change more broadly (e.g., dietary intake patterns), physical activity, eating disorders, and eating dysregulation across populations is beyond the scope of this review. However, research on these topics has been summarized elsewhere and is covered in several recent studies specific to individuals with overweight or obesity [16–21].

### ACT: A Brief Overview

ACT is an intervention approach that, like other so-called “third wave” or “third generation” therapies, uses mindfulness and acceptance strategies to foster behavior change and improve wellbeing [22, 23]. The overarching goal of ACT is to help individuals respond more flexibly to their thoughts, feelings, and physical sensations—sometimes referred to as “internal experiences”—so that they can better pursue living a life that is rich and personally meaningful.

One major focus of ACT is helping individuals to clarify their personal values and how these values can guide behavior. This focus is based on the premise that individuals’ wellbeing and health will be improved if individuals are engaging in actions aligned with values that are inherently meaningful and fulfilling, rather than allowing external benchmarks for success (e.g., a “goal weight”) or momentary thoughts and feelings (e.g., cravings) to dictate their behavior.

A second major focus of ACT is helping individuals to modify how they view and respond to their internal experiences so that they can pursue values-consistent behavior even when unwanted thoughts, feelings, or sensations are present. Traditional cognitive behavioral approaches focus on directly modifying and reducing unwanted thoughts and feelings. In contrast, ACT advocates for mindfully noticing and accepting these experiences as part of the human experience. This perspective is based on the tenets that efforts to directly control or change internal experiences are often unsuccessful, and that non-judgmental acceptance of such experiences more effectively allows individuals to re-direct their attention to constructive, values-consistent behavior. In ACT, individuals are taught a variety of mindfulness and acceptance skills to facilitate awareness and acceptance of internal experiences. Mindfulness skills are also often applied to increase individuals’ awareness of their values in daily life, as these can be difficult to keep in focus.

**Fig. 1:**
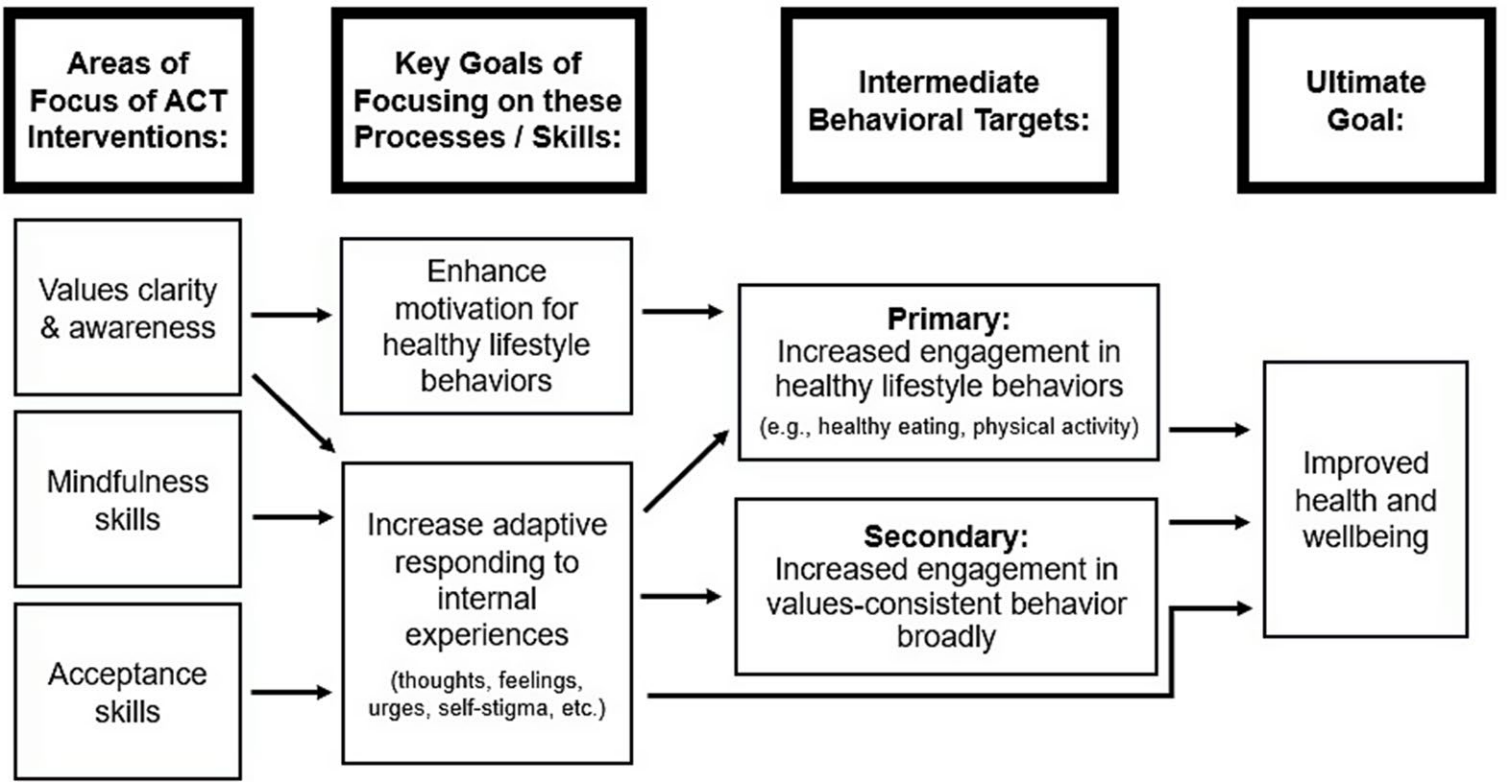
Key Components of ACT model as applied to obesity. Boxes with a thick border are section headers. Boxes with a thin border are model components. Arrows indicate hypothesized effects.

The exact ways in which ACT has been applied to obesity have varied between studies. However, many interventions have sought to (1) clarify and increase awareness of values, (2) clarify how lifestyle behaviors align with and can facilitate values-consistent living, with the hopes that doing so can enhance motivation to engage in these behaviors long-term, and (3) use mindfulness and acceptance skills to respond in adaptive ways to difficult internal experiences (e.g., cravings, negative thoughts, internalized weight stigma). Figure 1 provides a basic overview of key ACT processes as they relate to obesity-related outcomes.

To give brief examples of how these ACT skills might be applied clinically—for values, individuals may be guided in clarifying broad values that are important to them and then be encouraged to explore how engaging in health behaviors and being at a healthy weight can empower them in these areas. For example, an individual might state that they strongly value being an active, engaged parent (broad value). They may then note that they sleep better and have more energy to play with their children when exercising regularly, and that being at a healthy weight improves their mobility, enabling them to engage in a broader range of activities with their children (value-health link). One way that mindfulness skills can be applied is by encouraging individuals to practice pausing before, during, and after episodes of eating to have heightened awareness of various aspects of their internal experience, such as how much hedonic versus physiological hunger they are experiencing. An example of how acceptance might be cultivated is by asking individuals to notice behaviors that they are willing to engage in only under optimal conditions of internal experience, such as being willing to exercise after work only if they feel no anxiety about other tasks that also demand their time and attention. Individuals can then be encouraged to experiment with engaging in these behaviors even if the conditions are not optimal; for example, by increasing their willingness to sit with the anxiety they feel about the other tasks that are not getting done while they exercise. More detailed discussions of ACT’s theoretical fit with obesity and how skills can be applied clinically are provided in articles by Lillis et al. and Forman, Butryn, et al. [24–26]. For an overview of reviews and meta-analyses of ACT for obesity, see Table 1.

**Table 1:** Overview of Reviews / Meta-Analyses of ACT for obesity

**Foundational Review Papers**		
*Study*	*Key Areas of Focus / Strengths*	*Limitations*
Lillis & Kendra, 2014^24^	• Compares standard behavioral approaches with ACT, highlighting potential benefits and challenges of combining these methods • Reviews empirical evidence and models suggesting ACT may be effective as add-on or combined treatment	• Future research needed to determine whether adding ACT to standard treatment offers enough added value to justify extra training, time, and complexity involved
Forman & Butryn, 2015^26^	• Proposes acceptance-based, self-regulation model for long-term weight maintenance • Highlights role of hedonic drives and modern food environment in promoting unhealthy eating • Identifies key self-regulation skills: metacognitive awareness, distress tolerance, values clarity and behavioral commitment	• Limited research using real-time, ecologically valid methods to identify psychological processes driving eating and activity • Unclear which treatment components most effective • Lack of research on long-term outcomes
Forman et al., 2015.^25^	• Reviews rationale, current evidence, and gaps in ACT research for obesity • Concludes ACT effective for weight loss, especially in individuals highly reactive to internal/external eating cues • Highlights future research needs (e.g., study replication, long-term outcomes)	• Few RCTs and long-term studies limit conclusions about ACT’s effectiveness and moderating/mediating variables
**More Recent Reviews / Meta-Analyses**		
*Study*	*Key Areas of Focus / Strengths*	*Limitations*
Griffiths et al., 2018^55^	• Review of ACT’s impact on body dissatisfaction and internalized weight stigma • Concludes ACT shows promise for these outcomes, with improvements observed in 5 of 6 studies • Preliminary data suggest improvements mediated by ACT processes/constructs	• Findings limited by methodological challenges (e.g., sample size, no allocation concealment) • More and larger trials needed to establish efficacy • Limited diversity in samples
Lawlor et al., 2020^73^	• Systematic review and network meta-analysis of various third-wave therapies’ impact on body weight and psychological and physical health outcomes in adults with overweight/obesity • Of third-wave therapies, ACT demonstrates most consistent effectiveness • ACT only therapy with RCT evidence showing efficacy beyond 18 months	• Limited research maturity with small sample sizes and few high-quality studies • Lack of data beyond 12 months • Many studies with high risks of bias • Limited demographic reporting and diversity
Iturbe et al. 2022^63^	• Systematic review of ACT’s impact on psychological well-being and weight management in adults with overweight/obesity • Concludes ACT effective tool for promoting emotional well-being in this population, with improvements in > 70% of studies • Effectively targeted process variables or health behaviors related to weight management in 50% of studies	• Few RCTs • Inconsistent analysis methods (intent-to-treat vs. completer) • Lack of long-term follow-up data
Chew et al., 2023^34^	• First meta-analysis of ACT’s impact on weight, eating behaviors, and psychological outcomes in adults with overweight/obesity • Found ACT improved BMI but not body mass • ACT improved psychological flexibility and internalized weight stigma • Limited evidence of effectiveness in changing eating behaviors	• Larger, more rigorous RCTs needed to provide more precise estimates • Limited reporting of treatment fidelity and adherence measures • Limited search to English databases
Cox et al., 2024^40^	• Scoping review of acceptability, feasibility, and preliminary efficacy of ACT interventions for weight management among adolescents (ages 11–18) with overweight/obesity • Across 13 studies of 6 ACT interventions, high acceptability, strong attendance, and good retention • Some evidence that changes in key ACT processes support better self-regulation and weight change	• Low quality of evidence due to few studies, small sample sizes, limited study durations, etc. • Only three heterogeneous RCTs included
Pitil et al., 2024^72^	• First known review of ACT interventions specifically in RCTs targeting weight-related challenges in adults with overweight/obesity • Among included studies (*n* = 7), observed improvements in weight-related difficulties and percent weight loss in both ACT and non-ACT group • Concludes ACT promising intervention for addressing weight-related issues	• Some methodological limitations (e.g., lack of allocation concealment) • Potential lack of generalizability due to studies mostly focusing on adults and females from Western nations

Note. ACT = acceptance and commitment therapy; BMI = body mass index; RCT = randomized controlled trial

Additionally, many interventions have combined content from ACT with strategies from traditional behavioral modification interventions, such as stimulus control, self-monitoring, and goal setting [5]. Within the obesity literature, interventions using ACT are therefore often referred to as acceptance-based behavioral treatments, abbreviated ABT or ABBT. For simplicity across interventions with multiple foci and approaches, we have opted to use “ACT” or “ACT-based” herein, which encompasses ABT/ABBT.

### Foundational Studies of ACT for Obesity

Most early work on ACT for obesity, conducted in the mid-2000s to mid-2010s, focused on investigating ACT’s efficacy for weight loss relative to standard behavioral interventions. Building on promising pilot studies [27–29], results from several larger, randomized controlled trials showed that ACT-based interventions produced mean weight losses on par with or exceeding those from standard behavioral interventions [30–33].

For example, in one study, participants in an ACT-based intervention had greater weight loss relative to standard treatment at one year (M ± SD: 13.3% ± 0.83% in ACT vs. 9.8% ± 0.87% in standard) [32]. These differences had attenuated by the final follow-up at three years (4.7% ± 10.1% in ACT vs. 3.3% ± 8.2% in standard), although more individuals in the ACT-based intervention maintained ≥ 10% weight loss (a clinically significant target of such interventions) [31]. In another study among individuals with obesity and high internal disinhibition (i.e., susceptibility to eat in response to internal experiences), participants who received an ACT-based versus standard behavioral intervention had greater weight loss two years after starting treatment (M ± SE: 4.1% ± 0.88 in ACT vs. 2.4% ± 0.87 in standard) [33]. In another trial, mean weight loss at one year was comparable across ACT and non-ACT conditions (M ± SD: 10.84% ± 7.04% in ACT vs. 10.21% ± 7.98% in one non-ACT condition and 10.62% ± 7.82% in another). However, the ACT-based intervention was more efficacious for certain subgroups—here, individuals identifying as African American. Specifically, in the ACT condition, weight losses were similar for those identifying as African American and White—9.4% vs.11.5%, respectively–whereas greater differences in weight loss by race were observed for the non-ACT conditions [30].

Some of these studies also provided preliminary support that ACT could positively impact other outcomes of interest, such as internalized weight stigma, psychological distress, and quality of life [33, 34]. Additionally, a number of smaller studies investigated ACT’s efficacy for reducing weight recurrence and improving other aspects of health and wellbeing following metabolic bariatric surgery. On the whole, results were promising [35, 36]. For example, in one pilot study among patients who had undergone metabolic bariatric surgery and experienced ≥ 10% weight recurrence, weight recurrence was stopped and even reversed during a 10-week ACT intervention, with mean (± SD) weight losses of 3.58% ± 3.02% [36].

Overall, these seminal studies provided initial empirical support for ACT-based interventions as an alternative to standard behavioral treatment and raised the possibility of enhanced efficacy for certain outcomes or populations. Below, we provide an overview of more recent work in these areas.

### ACT for Weight Loss and Weight Loss Maintenance

#### New Populations

The influential studies on ACT discussed above focused on adults with overweight or obesity. One novel area of research has been to test ACT’s efficacy among adolescents or teenagers with overweight or obesity. Preliminary work with this population has demonstrated promising results for decreasing BMI [37–39]. For example, as highlighted in a 2023 scoping review of ACT for weight management among adolescents (Table 1), these studies have generally observed strong acceptability and feasibility of this approach, as well as decreases in BMI [40]. Given these preliminary findings, a fully powered randomized controlled trial is ongoing to test the efficacy of this treatment modality for this population [41].

#### New Settings and Efforts to Enhance Outcomes and Scalability

In most early trials of ACT for obesity, the interventionists were doctoral-level psychologists (or psychology trainees) with advanced training in ACT, and interventions were delivered in-person in research-oriented, academic settings. Several lines of research have recently explored how ACT-based interventions for obesity might be integrated into more “real world” or routine clinical settings by modifying one or more of these features. See Table 2.

**Table 2:** Studies evaluating impact on weight in novel contexts

Study	Novel Setting or Mode of Delivery	Impact of ACT Intervention on Weight Outcomes
Carels et al., 2019^50^	ACT group as a stepped-care supplement to self-help	Among those with low initial weight loss, stepped participants assigned to ACT and stepped participants continuing with self-help had similar weight loss at end of treatment (16 weeks).
Frayn et al. 2020^42^	Physician delivery	No significant weight loss in ACT or standard care conditions from pre- to post-treatment (8 sessions).
Bricker et al., 2021^43^	Telephone coaching	Percent of participants who reached ≥ 10% weight loss per scale-reported weight was as follows: • 3 months: 15% ACT, 4% SBT • 6 months: 24% ACT, 13% SBT • 12 months: 30% ACT, 30% SBT Greater 12-month weight loss was observed for ACT for self-reported and multiply imputed weights.
Lillis et al., 2021^45^	1-day workshop with telephone coaching and emails	ACT had significantly greater weight loss than control at 3, 6, 12, 18, and 24 months. Self-regulation (SR; additional non-ACT condition) had significantly greater weight loss than control at 3, 6, and 12 months. ACT and self-regulation had similar weight losses across time. Weight loss percentages were as follows: • 3 months: o ACT: 10.6% o SR: 10.5% o Control: 7.6% • 6 months: o ACT: 10.1% o SR: 9.6% o Control: 6.6% • 12 months: o ACT: 9.1% o SR: 7.8% o Control: 4.8% • 18 months: o ACT: 8.2% o SR: 6.0% o Control: 3.0% • 24 months: o ACT: 7.2% o SR: 4.2% o Control: 1.2% Evidence for greater weight loss in ACT among those with higher initial weight loss.
Levin et al., 2021^19^	Online guided self-help with telephone coaching	ACT had greater weight loss compared to waitlist control at end of treatment (8 weeks). Pre- to post-treatment weight loss in lbs.: • ACT: 5.87 lbs. • Waitlist: 2.09 lbs.
Mueller et al., 2022^47^	Online guided self-help with non-specialist telephone coaching	No significant difference in weight loss between ACT and standard advice waitlist control at end of treatment (4 months).
Potts et al., 2022^48^	Guided self-help book with telephone coaching or emails	No significant difference in BMI between ACT conditions (self-help + phone coaching, self-help + emails) and waitlist control at end of treatment (8 weeks).
Sherwood et al., 2022^49^	Individual, in-person ACT for suboptimal responders in an adaptive intervention	Among those with low initial weight loss, participants re-randomized to ACT and those re-randomized to portion controlled meals (non-ACT comparator) had similar weight loss at 6 and 18 months.

One approach has been to test the feasibility and efficacy of training physicians to deliver ACT-based interventions in primary care settings. Current data suggest this approach may be challenging, although only one study to-date has tested an ACT-based weight loss intervention delivered in this format. This study demonstrated no significant weight loss and high rates of attrition, with the authors suggesting that physicians do not have time to adequately deliver an ACT-based intervention [42].

Other studies have tested the viability of alternative treatment formats, such as telehealth, workshops, and web-based self-help, to enhance intervention scalability. One pilot study comparing an ACT-based telehealth treatment to standard behavioral telehealth treatment found that weight loss was greater in the ACT-based condition, although interpretation of results was limited by a lack of statistical power [43]. These authors are currently conducting a larger, fully powered version of this trial to determine the efficacy of this approach with greater confidence [44]. Another approach has been to deliver an ACT-based intervention in a workshop format, thus reducing interventionist contact time. A study testing this delivery format, which focused primarily on weight loss maintenance, also observed greater weight loss for the ACT versus the comparison intervention [45]. A larger, fully powered study of this approach is underway [46].

Several studies have tested self-help approaches with mixed outcomes. One study of an online, guided self-help intervention observed better weight loss for the ACT condition compared to control [19], while two other self-help interventions did not show significant differences in weight loss between the treatment and control conditions [47, 48]. Similarly, two studies examining the utility of an ACT-based intervention as a supplement to treatment for individuals experiencing minimal initial weight loss showed no differences between the ACT condition and comparator condition(s) [49, 50]. However, in at least one of these studies, findings may have been impacted by suboptimal participant engagement [48, 50].

Researchers are also actively testing which components of ACT-based interventions might optimize weight loss outcomes. This can inform treatment refinement and potentially lead to more streamlined and thus more easily scalable treatments. An ongoing trial is testing the independent and combined efficacy of each component of an ACT-based intervention on weight loss outcomes, with the goal of determining which combination of treatment components produces greater weight loss outcomes than standard behavioral treatment [51].

### ACT for Selected Obesity-related Outcomes Beyond Weight

#### Body-related Concerns & Internalized Weight Stigma

Body image dissatisfaction and internalized weight stigma are commonly reported challenges for people with obesity that may cause distress as well as undermine weight control efforts [11, 52, 53]. ACT has been posited to help individuals cope with and address these issues by fostering greater self-acceptance and psychological flexibility (i.e. the ability to be aware of and experience thoughts and feelings while responding flexibly to the circumstances and letting values guide behavior), including greater psychological flexibility specific to concerns around weight [24, 54].

A 2018 systematic review of studies utilizing ACT to target body image dissatisfaction and internalized weight stigma found that medium to large effect sizes were observed in 4 of 6 studies (several of which are discussed above), emphasizing that ACT shows promise in supporting individuals with obesity at developing more positive relationships with their bodies [55]. However, the review cautioned that additional research was needed given the limited number of studies included in the review, as well as methodological limitations of the studies that were included that may impact the validity of findings.

Since 2018, several additional studies have examined the effects of ACT-based interventions on body-related concerns and internalized weight stigma. One recent, large, randomized controlled trial evaluated the effects of a group-based ACT and mindfulness intervention on a range of physical and psychological outcomes among adults with elevated BMIs. This study found that participants receiving ACT reported lower impact of weight on quality of life than those in a treatment-as-usual control group [56]. Several randomized controlled trials of an ACT-based self-help intervention have found that this approach reduces internalized weight stigma among adults with obesity [19, 48, 57]. These findings highlight the adaptability of ACT for addressing body-related concerns and internalized weight stigma in various contexts.

#### Eating Regulation

Dysregulated eating habits, including emotional eating and binge eating, are common among people with obesity and make weight loss and sustained weight management challenging [58–60]. ACT has been theorized to help improve eating regulation by teaching individuals mindfulness and acceptance strategies that they can use to respond to their cravings and emotions in more adaptive ways. However, empirical data on ACT’s efficacy for improving eating regulation among adults with overweight or obesity are mixed.

For example, one randomized controlled trial found that an ACT-based group intervention reduced emotional eating and uncontrolled eating (which is an umbrella term used to capture difficulties in eating regulation and often includes loss of control overeating) among women with obesity compared to a treatment-as-usual group at post-treatment [61]. Another large, recent, randomized controlled trial found that ACT was more effective at reducing external eating (defined as eating in response to external food cues, regardless of internal signals of hunger and fullness) than a control group both at post-treatment and 6-month follow-up [56]. ACT was also shown to reduce binge eating among Veterans with overweight or obesity who reported difficulties with stress-related eating [62]. These findings and several others suggest that ACT may provide targeted benefits for specific eating behaviors [63–65]. However, a recent systematic review and meta-analysis, as well as a second systematic review, found that no significant differences were present across studies in emotional or uncontrolled eating at post-treatment or follow-up [34, 63]. These mixed results underscore the variability in eating-related outcomes currently assessed in the literature, and highlight the need for high-quality studies and consistent methodologies to better explore ACT’s role in eating regulation.

Despite these mixed findings, emerging studies continue to explore ACT’s potential in addressing eating regulation [66, 67]. Their findings, though limited by both sample size and study design, suggest ACT’s potential for addressing eating regulation challenges in diverse populations and settings. Further rigorous research is needed to confirm these effects and to determine their generalizability to other populations.

## Discussion

ACT is a psychotherapy intervention approach with strong data supporting its use for a wide range of mental and behavioral health concerns [68–71]. The literature base investigating ACT’s efficacy for obesity and obesity-related concerns remains more limited than research examining use of ACT for some other presenting problems, such as depression and anxiety [68]. Additionally, many studies have methodological limitations that must be considered, such as more limited sample sizes and recruitment of selective samples. However, several themes have emerged within the current evidence base.

First, for weight loss, while some early studies suggested that ACT produced weight losses superior to standard behavioral treatments, it may now be more accurate to view ACT as a potentially useful alternative or adjunct to standard behavioral treatment—one that in some cases produces superior weight loss and in others produces comparable weight loss on average. This observation aligns with the results of several recent systematic reviews or meta-analyses assessing ACT’s impact on weight [34, 63, 72, 73]. Importantly, very few studies have found that ACT produces *worse* weight loss than standard treatments. The efficacy of ACT might be attributed to a few factors. First, it is important to recognize that many versions of ACT-based behavioral weight management interventions devote a substantial amount of intervention time and focus to traditional behavioral skills, such as self-monitoring, goal setting, and stimulus control. Some or all of these traditional skills likely form an important foundation for learning acceptance-based skills. There is not yet a large enough body of literature evaluating the individual components of ACT to form a definitive conclusion about which components are most powerful [51]. In the clinical experience of the authors of this review, however, participants often find the acceptance components of the treatment to be the most novel and transformative, as it provides them with a mindset for approaching their weight control that feels fundamentally different than how they have previously attempted behavior change. Additional research is needed to clarify the factors that affect ACT’s relative efficacy (e.g., dose, format, delivery alongside other specific skills, interventionist training, target population) and for whom ACT may be most beneficial.

A somewhat mixed picture has emerged for ACT’s influence on facets of eating regulation like emotional eating [34, 63]. In contrast, while not all studies have reported on ACT’s effect on internalized weight stigma or body-related concerns, studies that have reported on these outcomes have tended to observe positive findings [34, 55, 63]. When ACT is integrated into behavioral weight management interventions, it is often delivered in a way that conveys tremendous empathy for the experience of attempting weight loss in the modern food environment, in which biologically based drives, such as hedonic hunger, are frequently and powerfully activated by the availability of highly processed foods engineered to be addictive. Individuals are encouraged to recognize the limits of being able to change or control such aspects of their internal experience. This may help them to reduce the extent to which they blame themselves for weight gain they have previously experienced. Several ACT skills also focus on viewing internal experiences more flexibly and decoupling these experiences from both behavior and one’s sense of self. These skills may help to reduce internalized weight stigma or the distress associated with it. In partial support of this hypothesis, ACT has been shown to reduce stigma or self-stigma related to a variety of domains beyond weight [74, 75]. More consistent measurement and reporting of internalized weight stigma and body-related concerns in ACT-based trials conducted across a range of settings, populations, intervention delivery formats, and research teams can help to clarify and confirm these effects.

There are several important directions for future research on ACT for obesity. One is to continue to explore the best pathways for disseminating ACT-based interventions to more routine care settings, whether that be through technology, self-help, training of or partnership with health providers to deliver interventions, or some other means. Several potential challenges need to be overcome to move ACT from primarily academic research settings to more routine care settings. These include challenges related to cost/coverage, who should serve as interventionists (if present) and how to train them to ensure treatment fidelity, and how to balance making ACT-based interventions easily disseminable with ensuring an adequate “dose” for effectiveness.

Future research should also continue to investigate which ACT concepts or skills provide the greatest benefit for different outcomes (e.g., weight, internalized weight stigma), and for whom an ACT approach may be more effective than a standard treatment approach. While several studies have evaluated potential treatment moderator/mediator effects and an optimization trial is currently under way, continued research on these topics may help with treatment matching and strategic streamlining of ACT-based treatments, respectively [24, 27, 51, 76]. Additionally, while behavioral treatment remains a recommended treatment for obesity, other interventions like metabolic bariatric surgery and obesity medications are also viable treatment options for many individuals [3, 4]. These approaches, especially medications, are also increasingly popular [77]. Future studies can examine how ACT might benefit patients with obesity utilizing one or more of these other intervention approaches; indeed, several trials of ACT among metabolic bariatric surgery populations are underway.

## Conclusions

In conclusion, the ACT model demonstrates a strong theoretical fit with many challenges faced by individuals with obesity, and data to date suggest that ACT produces similar or greater weight loss than standard behavioral interventions and may positively impact a variety of aspects of wellbeing, such as internalized weight stigma. Future research using rigorous research designs is needed to further clarify ACT’s impact on clinical outcomes of interest, to identify for whom and in what format ACT provides the greatest benefit, and to determine the best avenues for connecting individuals likely to benefit from ACT with these interventions in real-world settings.

**Key References**.

Papers of particular interest, published recently, have been highlighted as:

• Important reference.
•• Very important reference.

• Bricker JB, Mull KE, Sullivan BM, Forman EM, Lillis J, McTiernan A, et al. Telehealth acceptance and commitment therapy for weight loss: protocol of the WeLNES full scale randomized controlled trial. Contemp Clin Trials. 2023;126:107091. doi: 10.1016/j.cct.2023.107091.

Protocol overview of RCT comparing an ACT-based telehealth coaching intervention for weight loss to standard behavioral therapy in adults with overweight or obesity.

•• Chew HSJ, Chng S, Rajasegaran NN, Choy KH, Chong YY. Effectiveness of acceptance and commitment therapy on weight, eating behaviours and psychological outcomes: a systematic review and meta-analysis. Eat Weight Disord. 2023;28(1):6. doi: 10.1007/s40519-023-01535-6.

ACT improves psychological flexibility, reduces weight-related stigma, and reduces BMI, but shows limited effectiveness in changing other weight outcomes and eating behaviors.

•• Cox JS, Iturbe I, Searle A, Maiz E, Hinton EC. The acceptability, feasibility and preliminary efficacy of acceptance and commitment therapy for adolescents in the management of overweight or obesity: a scoping review. J Contextual Behav Sci. 2024;31:100713. doi: 10.1016/j.jcbs.2023.11.004.

ACT for weight management in adolescents (aged 11–18) with overweight and obesity appears feasible and acceptable and shows promise for improving psychological flexibility, self-regulation, and potentially weight, aligning with research among adults.

•• Iturbe I, Echeburúa E, Maiz E. The effectiveness of acceptance and commitment therapy upon weight management and psychological well-being of adults with overweight or obesity: A systematic review. Clin Psychol Psychother. 2022;29(3):837 − 56. doi: 10.1002/cpp.2695.

Provides a comprehensive review of the effectiveness of ACT for promoting psychological well-being in adults with obesity or overweight.

• Manasse SM, Moussaoui JR, Lampe EW, Brown KL, Zhang F, Janicke DM, et al. Evaluating an acceptance-based lifestyle modification program to address cardiovascular disease risk among adolescent girls with overweight and obesity: protocol for a randomized controlled trial. Contemp Clin Trials. 2024; 144:107634. doi: 10.1016/j.cct.2024.107634.

Protocol overview of an ACT intervention for adolescent girls with overweight or obesity to test the effect of ACT on cardiometabolic health, health-related behaviors, and psychological factors in a fully powered trial.

•• Pitil PP, Ghazali SR. Acceptance and commitment therapy and weight-related difficulties in overweight and obese adults: a systematic review. Psychol Rep. 2024;127(6):2873-97. doi: 10.1177/00332941221149172.

Supports ACT as an effective intervention for adults with overweight and obesity to address weight-related difficulties and excess body weight.

## Data Availability

No datasets were generated or analysed during the current study.
